# Epidemiology, Clinical Manifestations, Treatment, and Outcome of Mucormycosis: A Review of 77 Cases From a Single Center in France

**DOI:** 10.1093/ofid/ofae426

**Published:** 2024-07-23

**Authors:** Blandine Denis, Matthieu Resche-Rigon, Emmanuel Raffoux, Anne-Marie Ronchetti, Emmanuel Dudoignon, Benjamin Verillaud, Sandrine Valade, Gwenaël Lorillon, Florence Rabian, Aliénor Xhaard, Sophie Touratier, Samia Hamane, Alexandre Alanio, Nathalie De Castro

**Affiliations:** Department of Infectious Diseases, Hôpital Saint-Louis, Assistance Publique–Hôpitaux de Paris, Paris, France; Department of Biomedical Statistics and Methodology, Hôpital Saint-Louis, Fernand Widal, Lariboisière, Assistance Publique–Hôpitaux de Paris, Paris, France; Université Paris Cité, Paris, France; Université Paris Cité, Paris, France; Department of Hematology, Hôpital Saint-Louis, Fernand Widal, Lariboisière, Assistance Publique–Hôpitaux de Paris, Paris, France; Department of Hematology, Hôpital Saint-Louis, Fernand Widal, Lariboisière, Assistance Publique–Hôpitaux de Paris, Paris, France; Université Paris Cité, Paris, France; Department of Burn Intensive Care Unit, Hôpital Saint-Louis, Assistance Publique–Hôpitaux de Paris, Paris, France; Department of Head and Neck surgery, Hôpital Lariboisière, Assistance Publique–Hôpitaux de Paris, Inserm U1131, Université Paris Cité, Paris, France; Department of Intensive Care Medicine, Hôpital Saint-Louis, Fernand Widal, Lariboisière, Assistance Publique–Hôpitaux de Paris, Paris, France; Department of Pneumology, Hôpital Saint-Louis, Assistance Publique–Hôpitaux de Paris, Paris, France; Department of Hematology–Teenagers and Young Adults Unit, Hôpital Saint-Louis, Assistance Publique–Hôpitaux de Paris, Paris, France; Service d’hematologie–greffes, Hôpital Saint-Louis, Assistance Publique–Hôpitaux de Paris, Paris, France; Department of Pharmacy, Hôpital Saint-Louis, Assistance Publique–Hôpitaux de Paris, Paris, France; Laboratoire de parasitologie-mycologie, Hôpital Saint-Louis, Assistance Publique–Hôpitaux de Paris, Paris, France; Laboratoire de parasitologie-mycologie, Hôpital Saint-Louis, Assistance Publique–Hôpitaux de Paris, Paris, France; Département de mycologie, Institut Pasteur, Université Paris Cité, Centre National de Référence Mycoses Invasives et Antifongiques, Groupe de recherche Mycologie Translationnelle Paris, France; Department of Infectious Diseases, Hôpital Saint-Louis, Assistance Publique–Hôpitaux de Paris, Paris, France; Université Paris Cité, Paris, France

**Keywords:** diagnosis, epidemiology, immunosuppressed, mucormycosis, outcome

## Abstract

**Background:**

The aim of this study was to assess the epidemiology, clinical manifestations, and outcome of mucormycosis over 15 years in a single center in France.

**Methods:**

We conducted a retrospective analysis of all mucormycosis cases in our institution from 1 January 2006 to 31 December 2020 and analyzed patients’ medical records, laboratory results, and treatment to describe the epidemiology, clinical manifestations, diagnosis, treatment, and outcome. Mucorales quantitative polymerase chain reaction (qPCR) for the diagnosis was implemented in 2015.

**Results:**

Seventy-seven mucormycosis cases were analyzed in 77 patients, with a median age of 54 years (60% male). Identified risk factors were hematological diseases (46 cases [60%]), solid malignancies (2 cases), solid organ transplants (3), burns (18), diabetes only (7), and trauma (1). Sites of infection were lungs (42%), sinus (36%), skin (31%), central nervous system (9%), liver (8%), others (6%), and disseminated (12%). Diagnosis remained difficult and qPCR contributed to mucormycosis diagnosis in 30% of cases. Among hematology patients, serum qPCR was the only positive test in 15% of cases. A mixed mold infection was diagnosed in 24 of 77 (31%) patients. Surgical treatment was undertaken in 43 (56%) cases. Most patients received liposomal amphotericin B (89%), with a combination therapy in 18 of 77 cases (23%). Three-month survival rate was 40% (95% confidence interval [CI], .30–.53]). As for treatment, adjunction of surgery (hazard ratio, 0.47 [95%CI, .25–.91); *P* = 0.02) was associated with lower mortality.

**Conclusions:**

Mucormycosis remained associated with high mortality, especially in the hematological and burn populations. Surgery in combination with antifungal treatment was associated with improved survival.

Over the past 2 decades, mucormycosis has emerged as a major fungal infection with a high mortality rate overall, especially in patients with hematological malignancies (HMs) or with severe burn injury, who have a high 30-day mortality rate of 38% to 55% [1–4]. While in high-resource countries, the disease is mostly observed in patients with diabetes mellitus and hematological malignancies undergoing chemotherapy and allogeneic hematopoietic stem cell transplantation (HSCT), in low- and middle-income countries, mucormycosis cases mainly occur in patients with uncontrolled diabetes or trauma [5–9]. In 2005–2007, an analysis in France revealed a total of 101 cases of mucormycosis, mostly in men (58%), with 50% of cases occurring in patients with HM, 23% in patients with diabetes, and 18% in patients with trauma, with a 3-month mortality of 56% [10]. More recently, a review of 851 published cases between 2000 and 2017 confirmed previous data, with a 46% mortality rate, more disseminated infections for hematology patients, more rhino-orbital-cerebral mucormycosis (ROCM) among patients with diabetes, more *Rhizopus* species in diabetes cases, and higher mortality for *Cunninghamella* [2].

Assistance Publique–Hôpitaux de Paris (AP-HP) Saint-Louis Hospital is a tertiary hospital located in Paris with 9 hematology and oncology departments and a burn unit, treating patients at high risk of developing mucormycosis. We wished to describe the epidemiology, clinical, and microbiological presentation of mucormycosis over a 15-year period and assess treatment outcomes in the context of a highly specialized center.

## METHODS

### Study Design and Population

The study was a single-center retrospective analysis of all consecutive cases of invasive mucormycosis diagnosed at Saint-Louis Hospital from 1 January 2006 through 31 December 2020. Some cases have previously been published [11, 12].

This was a noninterventional study; no additional medical procedure was performed, and all data were retrieved from the medical charts of the treated patients.

### Patient Consent Statement

The study was conducted in accordance with French ethical and approval regulations in a “hors loi Jardé” legislative context (https://www.legifrance.gouv.fr/codes/article_lc/LEGIARTI000045629992/2022–04–22) Patients treated at AP-HP Saint-Louis Hospital are informed that data extracted from their electronic health records may be used for noninterventional studies without requiring further consent, unless they manifest their opposition. Ethical approval was waived and the present study was not submitted for further ethics evaluation.

### Study Procedures

Data retrieved from patients’ medical and pharmacy electronic health records included age, sex, underlying condition and its treatment (list of all variables in Supplementary Appendix 1), patient signs and symptoms and radiological imaging at mucormycosis diagnosis as per European Organization for Research and Treatment of Cancer/Mycosis Study Group (EORTC/MSG) 2019 criteria (Supplementary Appendix 2), mucormycosis location, type of mycological diagnosis (direct examination, culture, quantitative polymerase chain reaction [qPCR]), signs of invasion on pathology examination, and presence of a concurrent fungal or bacterial infection. Mucormycosis treatment (type, dose, and duration for all antifungal treatments, surgical treatments, other measures (tapering of immunosuppressive treatment, treatment of diabetes), and clinical outcomes (overall and 3-month survival, mucormycosis relapse, refractory fungal infection, relapse of hematological disease, incidence of severe complications, and cause of death, if concerned) were also retrieved.

Of note, the Mucorales qPCR assay [13] was implemented in 2015 in our center and used as a targeted diagnosis approach and also as a screening method in high-risk burn patients with deep burn total body surface area above 20%. For those high-risk burn patients, the screening strategy consists of a twice-weekly Mucorales qPCR screening of the plasma. If the latter becomes positive, then a qPCR-guided preemptive treatment strategy is initiated with liposomal amphotericin B. Tissues suspected of fungal infection are excised, biopsies are sent to mycology laboratory, and, if all are negative, preemptive treatment stopped after 2 negative PCR results.

Clinical samples included respiratory samples (sputum, bronchoalveolar lavage, pleural fluid, pulmonary and tracheobronchial biopsies); pericardial biopsies; sinus/mastoidal biopsies; cutaneous lesions (swabs, biopsies); liver, colon, and kidney biopsies; and serum. Those samples were sent to the mycology laboratory for direct microscopic examination, culture, and molecular qPCR tests and to the pathology laboratory (see Supplementary Appendix 1 for full laboratory procedures).

### Outcomes and Definitions

Our primary endpoint was 3-month survival after mucormycosis diagnosis. Secondary endpoints included survival at the date of last follow-up or administrative censoring at 1 year, mortality related to mucormycosis, and causes of death.

Definition of mucormycosis cases are as follows:

- All cases were validated by a multidisciplinary committee (mycologists, infectious diseases physician, and pharmacist). We aimed to describe all our validated mucormycosis cases, including those made by qPCR in serum, even though serum qPCR is not yet included in the updated definitions of invasive fungal disease of the EORTC/MSG (Supplementary Appendix 2) [14]. Also, patients with burn injury and patients with diabetes, 2 well-recognized populations at increased risk of mucormycosis, are not considered as a host factor according the EORTC/MSG criteria, leading to underestimation of EORTC/MSG probable mucormycosis cases in those settings. We decided, in this report, to provide detailed descriptions of underlying conditions and radiological and microbial data and to classify, when possible, our validated cases according to EORTC/MSG criteria. Validated mucormycosis cases that did not correspond to EORTC/MSG criteria were classified as putative mucormycosis.
- Histological diagnosis of mucormycosis was defined as histopathology showing nonseptate, large ribbon-like hyphae, with vessel occlusion. If histology was completed by identification of mucormycosis by culture or qPCR on tissue, the case was defined as proven mucormycosis. If the latter culture or qPCR on tissue identification were absent, the case was classified as proven invasive fungal infection (IFI) suggesting mucormycosis.
- Disseminated mucormycosis was defined as identification of Mucorales in >2 noncontiguous sites (sinus and pulmonary, or sinus and orbital, for example, were not considered as disseminated disease).

Treatment used within the first 15 days was considered effective if it included liposomal amphotericin B or posaconazole or isavuconazole. Monotherapy with echinocandins (caspofungin, micafungin) was considered noneffective. Treatments were classified as effective monotherapy, effective combination therapy, or noneffective/not treated.

### Statistical Analyses

Continuous variables are summarized as median and interquartile range (IQR) and categorical variables as counts and percentages. Evolution of the number of cases diagnosed was compared over 2 periods (before and after 2013) using a Poisson model and comparison among hematology patients with a Fisher test. Survival was defined as the delay between the date of mucormycosis diagnosis and the date of death. Survivals were estimated using the Kaplan-Meier method with censoring at the date of last follow-up or administrative censoring at 1 year. Associations between survival at 3 months and risk factors or treatment were assessed using Cox proportional hazard models. Hazard ratios (HRs) were estimated with their 95% confidence intervals (CIs) and tested using the Wald test. An analysis of 3-month mortality regarding effective treatment after mucormycosis diagnosis, in particular monotherapy versus combination therapy and the role of surgery, adjusted on effective antifungal treatment, was also undertaken.

All reported *P* values are 2-sided, and *P* values of <.05 were considered to indicate statistical significance. Analyses were performed with R software, version 3.4.1.

## RESULTS

**Figure 1.**
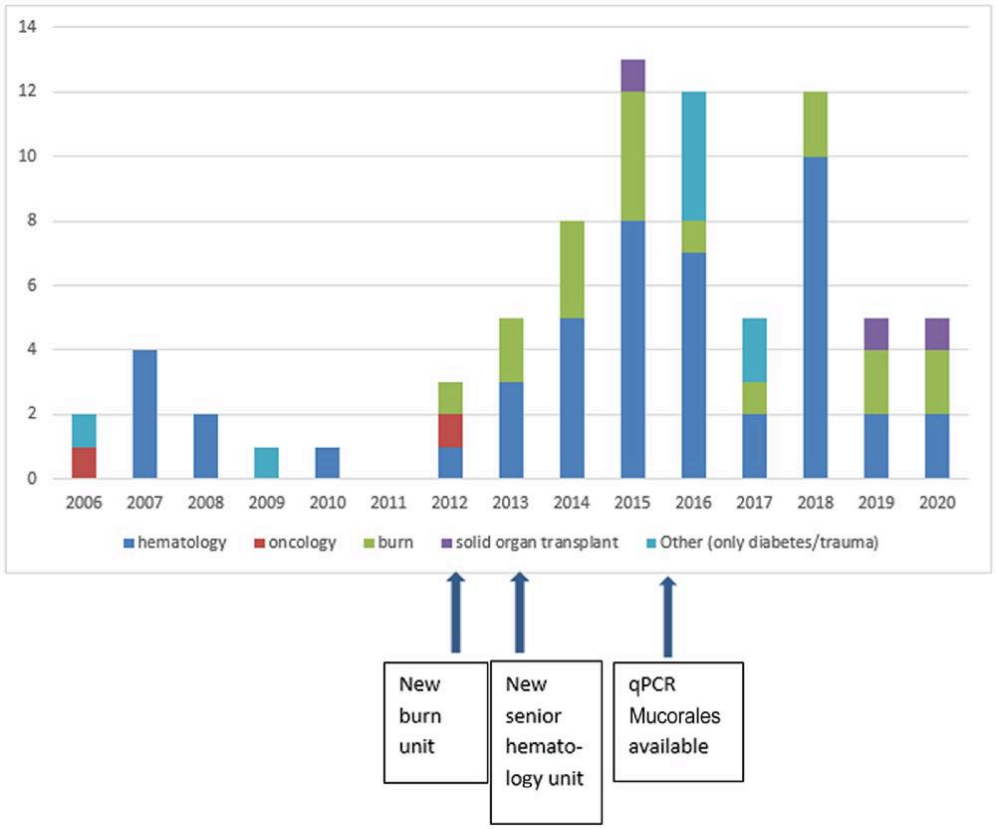
Number of mucormycosis cases reported per year at Saint-Louis Hospital between 1 January 2006 and 31 December 2020. Abbreviation: qPCR, quantitative polymerase chain reaction.

Over the study period, 77 mucormycosis cases were diagnosed in our center. The prevalence over time is shown in Figure 1. The majority of cases (n = 59 [77%]) were observed after 2013 (*P* = 0.001). Forty-six of 77 cases (60%) were observed in patients with HM, including 11 (14%) and 35 (46%) before and after 2013, respectively (*P* = 1). Among the 77 validated cases, 57 could be classified according to EORTC/MSG classification with 40 proven mucormycosis, 5 proven IFI suggesting mucormycosis, and 12 probable mucormycosis. Twenty cases either did not have a host factor (patients with diabetes, burns, or solid cancer) or had a diagnosis made by qPCR without biopsy and were classified as putative mucormycosis (Supplementary Table 1).

The characteristics of the patients at mucormycosis diagnosis are summarized in Table 1. Most patients (60%) were male, with a median age of 54 years (IQR, 35–69 years). Neutropenia (neutrophil count <500 cells/μL) was noted for 30 (39%) patients; 19 (25%) had diabetes, with, when available, a median glycosylated hemoglobin of 11%, with 7 (9%) having diabetes as the only risk factor for mucormycosis.

**Table 1.** Baseline Characteristics of the 77 Mucormycosis Cases

Underlying Conditions	All Patients (n = 77 [100%])	Hematology Patients (n = 46 [60%])	Oncology (Solid Tumor) Patients (n = 2 [3%])	SOT^a^ (n = 3 [4%])	Burn Patients (n = 18 [24%])	Diabetes Only (n = 7 [9%])	Trauma (n = 1 [1%])
Male sex	46 (60)	25 (54)	2 (100)	3 (100)	11 (61)	4 (57)	1 (100)
Age, y, median (IQR)	54 (35–69)	56 (35–70)	68 (66–70)	66 (63–70)	44 (34–54)	66 (52–71)	50
Underlying condition							
Immunosuppressive treatment	34 (44)	31 (67)	0	3 (100)	0	0	0
Neutropenia (<500 cells/μL)	30 (39)	29 (63)	0	1 (33)	0	0	0
Diabetes mellitus	19 (25)	11 (23)	0	1 (33)	0	7 (100)	0
Allogeneic HSCT recipient	20 (26)	20 (43)	0	0	0	0	0
Prior exposure to antifungals	39 (51)	33 (72)	0	1	3	2	0
Active against Mucorales	25 (35)	21 (46)	0	1	1	2	0
Location^b^							
Pulmonary/tracheobronchial (n = 36/42) abnormal CT scan	32 (42)	29 (63)	1 (50)	0	0	2	0
1 nodule	8 (19)	8 (22)	0	0	0	0	0
Multiple nodules	16 (38)	15 (41)	0	0	0	1	0
Halo sign	11 (26)	11 (30)	0	0	0	0	0
Consolidation	17 (40)	14 (38)	1	0	0	2	0
Others	12 (29)	10 (27)	0	1	0	1	0
ROCM^c^	28 (36)	18 (39)	0	2	1	7 (100)	0
Cutaneous	24 (31)	4 (9)	0	1	18 (100)	0	1 (100)
Liver	6 (8)	5 (11)	1	0	0	0	0
Spleen	4 (5)	4 (9)	0	0	0	0	0
Kidney	1 (1)	1 (2)	0	0	0	0	
Disseminated^d^	9 (12)	6 (13)	0	1 (33)	1 (6)	1 (14)	0
Coinfections							
Mixed infection with molds^e^	24 (31)	16 (35)	1	0	7 (39)	0	0

Data are presented as No. (%) unless otherwise indicated.

Abbreviations: CT, computed tomography; HSCT, hematopoietic stem cell transplant; IQR, interquartile range; ROCM, rhino-orbital-cerebral mucormycosis; SOT, solid organ transplant.

^a^Two liver transplant recipients and 1 kidney transplant recipient.

^b^One or more sites per patient possible.

^c^Sinus location: n = 28, with orbital extension in 3 cases and intracranial extension in 6 cases; central nervous system: n = 7 (6 intracranial extension from sinus + 1 disseminated case without ROCM).

^d^Two or more noncontiguous sites.

^e^Mixed mold infections: 16 among hematology patients: *Aspergillus* species in 15 cases and 1 *Alternaria*; 7 among patients with burn injury: 4 cutaneous fusariosis, 1 cutaneous aspergillosis, 1 with both cutaneous fusariosis and aspergillosis, and 1 with both cutaneous fusariosis and *Scedosporium*; 1 patient with esophageal cancer: *Aspergillus* species.

Among the 46 (60%) patients with HM, the majority had acute leukemia (26/46 [57%]), myelodysplastic syndrome (8/46 [17%]), and lymphoma (6/46 [13%]). Twenty (43%) had received an allogeneic HSCT, and 12 of 20 (60%) had developed graft-versus-host disease. Neutropenia was noted for 29 (63%) patients, 11 (24%) had diabetes, and 31 (67%) had an immunosuppressive treatment, with corticosteroids at mucormycosis diagnosis in 25 cases (54%) and with a corticosteroid dose >0.3 mg/kg for >3 weeks in 17 cases (37%). At mucormycosis diagnosis, 29 of 46 (63%) HM patients had a refractory hematological disease.

The majority of the 31 patients (40%) who did not have HM had thermal burns (18/31), with a median total burn surface area of 58% (IQR, 37%–69%).

Prior exposure to anti-mold antifungals before mucormycosis diagnosis was noted for 39 (51%) patients; in particular, 21 (28%) had received posaconazole, 19 (25%) voriconazole, 5 isavuconazole (7%), and 12 (16%) liposomal amphotericin B.

Main sites of infection were lungs/tracheobronchial (n = 32 [42%]). Results of the 42 pulmonary computed tomography (CT) scans reviewed are shown in Table 1: 36 CT scans showed abnormalities, mainly with consolidations (17/42 [40%]), multiple nodules (16/42 [38%]), halo sign (11/42 [26%]), and reverse halo sign (3/11). Only hematology patients had nodules with halo and/or reverse halo sign. Twenty-eight patients (36%) had ROCM with orbital extension in 3 cases and intracranial extension in 6 cases. Skin location was observed in 24 cases (31%). Two-thirds of the 9 (12%) disseminated cases occurred in hematology patients. One patient with disseminated disease had an intracranial localization without sinonasal involvement. Spleen infection (n = 4 [5%]) occurred among disseminated cases.

### Microbial and Histological Diagnosis of Mucormycosis

Mucormycosis identification by culture and/or qPCR was obtained in 72 of 77 (94%) cases; the latter 5 patients had a proven IFI suggesting mucormycosis on biopsies but with negative culture and unavailable molecular identification (Supplementary Table 2). For 28 of 77 (36%) patients, the culture was negative or unavailable: 5 patients had a proven IFI suggesting mucormycosis, and for the other 23 diagnoses, genus identification was possible by qPCR (with or without direct examination), accounting for 30% of cases (Supplementary Table 1). Diagnosis was made by a positive serum qPCR only in 10 cases (13%); in these cases, qPCR was the only positive test as direct examination was negative.

Focusing on the 46 hematology patients, 27 had at least 1 result of serum qPCR, with 22 of 27 (81%) patients with a positive serum qPCR. In 7 (15%) cases, serum qPCR was the only positive result.

A concomitant fungal or bacterial infection was observed in 36 cases (47%), including coinfection with another mold in 24 cases (31%) (Table 1), with yeasts in 2 cases (3%) (1 candidemia and 1 *Trichosporon* fungemia), and with bacteria in 10 cases (13%) (including 4 bacteremias, 6 mixed invasive sinusitis, and 1 pulmonary infection).

### Mucormycosis Treatment

Sixty-nine (90%) patients received liposomal amphotericin B at a median dose of 5 mg/kg/day, (IQR, 3–10 mg/kg/day), for a median length of 25 days (IQR, 7–53 days). Forty-eight (62%) patients received an azole, including posaconazole in 32 cases (42%) and isavuconazole in 16 (21%). Fifteen patients (19%) received caspofungin, in combination therapy in 11 of 15 cases. Regarding effective treatment during the first 15 days after diagnosis, 53 (69%) received effective monotherapy, 19 effective combination therapy (18 [23%] bitherapy, 1 [1.3%] tritherapy) and 5 (6.5%) were not treated/not effective. Among patients under immunosuppressive regimen, 13 of 34 (38%) had a tapering of immunosuppressive treatment.

Surgical treatment was performed in 43 (56%) patients.

**Figure 2.**
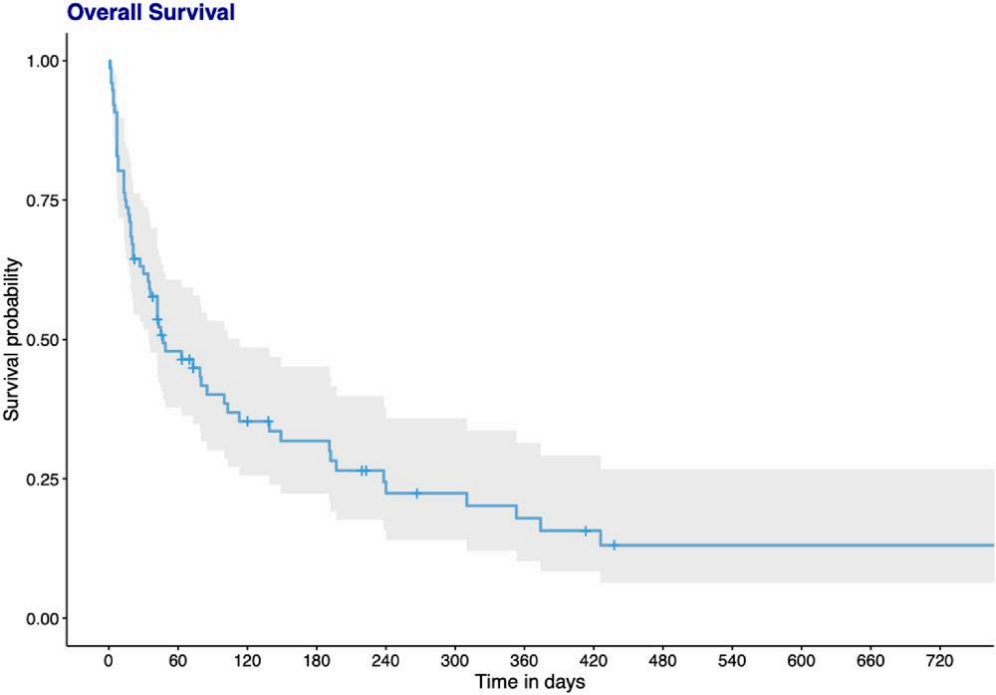
Overall survival after mucormycosis diagnosis.

**Figure 3.**
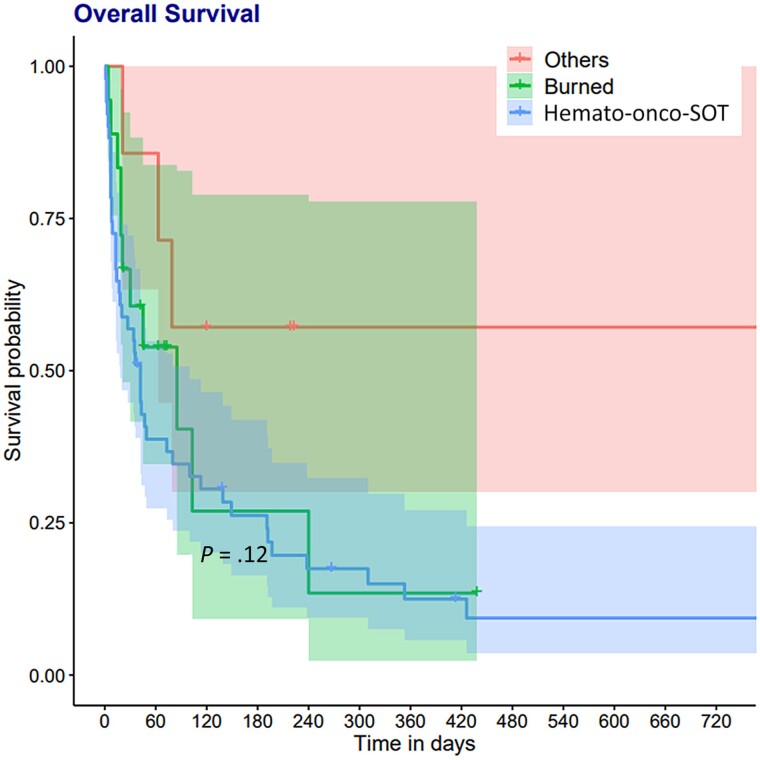
Differences between hematologic–oncologic–solid organ transplantation (hemato-onco-SOT)/burn/other patients on overall survival after mucormycosis diagnosis.

The 3-month survival after mucormycosis diagnosis was 40% (95% CI, .30–.53). Overall survival was 25% with 43% mortality related to mucormycosis. Overall survival and comparisons between hemato-oncologic–solid organ transplant, burn, and diabetes only–trauma patients are shown in Figures 2 and 3. There was no significant differences between the 3 groups in terms of survival (*P* = 0.12).

Looking at 3-month mortality risk factors, age at diagnosis (HR, 1.02 [95% CI, 1.0–1.04; *P* = 0.017) and having disseminated disease (HR, 2.84 [95% CI, 1.25–6.43]; *P* = 0.009) were significantly associated with 3-month mortality, whereas only surgical treatment (HR, 0.47 [95% CI, .25-.91); *P* = 0.02) was associated with survival in multivariate analysis. Surgery remained significantly associated with survival after adjustment on effective (monotherapy or combination therapy) antifungal treatment (HR, 0.41 [95% CI, .21–.82]; *P* = 0.01) (Table 2). No difference was observed between monotherapy and combination of effective treatments (*P* = 0.14).

**Table 2.** Baseline Risk Factors Associated With 3-Month Mortality and Impact of Mucormycosis Treatment on Outcome

Variable	Univariate Analysis		Multivariate Analysis	
	HR (95% CI)	*P* Value	HR (95% CI)	*P* Value
Male sex	0.83 (.46–1.5)	.54	…	
Age	1.02 (1.0–1.04)	.017	1.01 (1.0–1.03)	.11
Diabetes only + trauma (reference, n = 8)	1		…	
Hemopathy-oncology-SOT (n = 51)	2.28 (.70–7.45)	.17	…	
Burn injury (n = 18)	1.61 (.44–5.97)	.47	…	
Pulmonary mucormycosis	1.44 (.8–2.6)	.22	…	
Sinus	0.89 (.48–1.64)	.71	…	
Skin location	0.99 (.54–1.84)	.98	…	
Other site	1.08 (.6–1.93)	.81	…	
Disseminated mucormycosis	2.84 (1.25–6.43)	.009	1.86 (.8–4.33)	.15
Impact of mucormycosis treatment on 3-mo mortality				
Tapering immunosuppressive regimen (n = 13/34)	0.78 (.35–1.76)	.55	…	
Surgical debridement (n = 43)	0.4 (.22–.73)	.002	0.47 (.25–.91)	.02
Surgical debridement adjusted on effective treatment (monotherapy or combination therapy)	0.41 (.21–.82)	.01	…	

Abbreviations: CI, confidence interval; HR, hazard ratio; SOT, solid organ transplant recipient.

## DISCUSSION

Our study gives an overview of the epidemiology of mucormycosis in our highly specialized center, with an increasing number of cases declared over a period of 15 years, including 80% of cases observed between 2013 and 2020. Most patients had HM, followed by patients with burn injury and diabetic patients. Two-thirds of patients with HM had a refractory HM disease at mucormycosis diagnosis, with some patients cumulating fungal risk factors such as neutropenia, corticosteroid use, immunosuppressive regimen, and diabetes. Diagnosis remains difficult, still relying mainly on biopsies, but qPCR DNA Mucorales is a sensitive diagnostic tool, improving diagnosis in 30% of cases in our study. Of note, a mixed mold infection was diagnosed in 24 of 77 (31%) patients. Regarding treatment, surgical treatment was protective of death but not combination therapy, and mortality remains high, especially in patients with HM and burn injury.

Our retrospective study illustrates the evolving epidemiology of invasive fungal diseases, with 80% of cases diagnosed in the most recent period. Comparing with European publications before our study period, the proportion of patients with HM is higher [9, 10], and most of the mucormycosis diagnoses were performed in patients cumulating risks factors for IFI and/or with refractory diseases. Since 2021, most mucormycosis cases published were among patients with coronavirus disease 2019 (COVID-19), showing that among 341 patients from Asian countries and the United States in the MUNCO (Mucormycosis in COVID-19) registry, diabetes was the most common risk factor (85% of cases) [15], while in the French COVID/mucormycosis cohort, HM was present in 35% with a 3-month mortality rate of 88% [16]. In our study, the increase of mucormycosis diagnoses is also partially explained by, first, the arrival in our institution of a burn unit department in 2012 and a senior hematology department in 2013, and second, by the availability of Mucorales qPCR as a new sensitive diagnostic tool since 2015.

Mucorales grow uneasily, and, before the availability qPCR as a diagnostic tool, direct examination and/or culture were the main diagnostic criteria. The qPCR assay can be used in plasma/serum, on biopsies, or on respiratory fluids and has greatly improved mucormycosis diagnosis [17, 18-19]). It allows earlier antifungal initiation when positive in plasma, with a sensitivity of 85% and a specificity of 90% [17]. This qPCR is now used in some French centers as a screening strategy in addition to *Aspergillus* qPCR and galactomannan. In our study, a diagnosis was established by qPCR on serum and/or biopsies for 23 of the 28 patients with negative or unavailable cultures, and qPCR was the only positive examination in 13% of cases. Also, among hematology patients, serum qPCR was the only positive examination in 15% of cases. Since most patients had refractory hematological diseases, with invasive diagnostic methods difficult to implement, making a diagnosis with a serum test is a real improvement. qPCR is also helpful in cases of mixed infections, since other fungi grow more easily and can restrict Mucorales growth. It is also noteworthy that a mixed mold infection was noted in 24 of 77 (31%) cases, especially with *Aspergillus* species in patients with HM and mainly with *Fusarium* species in patients with burn injury. Accurate microbial diagnosis is essential since occultation of mucormycosis in a mixed mold infection could lead to an inefficient voriconazole prescription.

In our study, liposomal amphotericin B and posaconazole were mainly used as curative treatment, and surgery was protective of death. There were too few cases to estimate the effect of tapering of immunosuppressive regimen and the effect of isavuconazole. The use of caspofungin or posaconazole as adjunctive treatment had no effect on mortality, but the number of cases were low. Also, since it is a retrospective study, treatment allocation bias according to severity might have happened. Management of mucormycosis is still being debated. International societies recommend surgery, when feasible, and first-line monotherapy with a high dose of liposomal amphotericin B [20,21,22,25,26]. Since the disease is rare, there are no clinical trials in humans comparing different daily doses of liposomal amphotericin B, nor combination therapies versus monotherapies; thus, practices are quite heterogeneous around the world. Mortality remains high in the hematology and burn populations and has not improved since the last national French survey during the 2005–2007 period [10]. In our study, mucormycosis appeared to be mainly an “end-stage” manifestation for the hematology patients, with two-thirds having a refractory HM at mucormycosis diagnosis, and mucormycosis diagnosed under posaconazole prophylaxis in 28% of cases. Thus, mortality directly related to mucormycosis is difficult to assess in this context. For patients with burn injury, a median burn surface ≥30% is considered severe, with at least a 30% mortality rate at this level, and mortality increases rapidly with the percentage increase of burn surface. Among our cases, median burn surface was 58% (IQR, 37%–69%), at least partly explaining the high mortality rate observed.

Our study has some limitations. First, it is a monocentric retrospective study, with only 77 cases and with a subgroup analysis of patients with hematological malignancies–oncology–SOT, patients with burn injury, and others. Second, the number of cases is too small to study the effect of tapering of immunosuppressive regimens and the effect of isavuconazole, and we did not study the effect of early versus delayed treatment on mortality. However, with 77 cases of a very rare disease with an estimated prevalence of 1.2 million in France in 2006 [23], this study is a large series on mucormycosis. The fact that it is monocentric also facilitates comparison over the study period since clinical, diagnostic, and therapeutic management are more homogenous.

In conclusion, mucormycosis has emerged as an important IFI with severe mortality despite new diagnostic tools. In our study, mucormycosis was mostly observed in patients with refractory HM disease and patients with severe burns. New strategies need to be implemented to improve mucormycosis diagnosis and outcomes. The twice-weekly plasma qPCR screening strategy is now implemented in our burn unit department for patients with a median burn surface ≥20%, and we still need to optimize early diagnosis and treatment in patients with HM, especially those who cumulate multiple IFI risk factors. A screening strategy could be interesting in that setting and would need to be further investigated.

## Supplementary Material

ofae426_Supplementary_Data

## Supplementary Data

Supplementary materials are available at *Open Forum Infectious Diseases* online. Consisting of data provided by the authors to benefit the reader, the posted materials are not copyedited and are the sole responsibility of the authors, so questions or comments should be addressed to the corresponding author.

## Notes

***Acknowledgments.*** The authors thank Professor Jean-Michel Molina for his support and advice during this study.

***Author contributions.*** B. D., M. R.-R., and A.-M. R. conceived and designed the study. B. D., M. R.-R., A. A., S. T., S. H., and N. d. C. contributed to interpretation of the results. M. R.-R. did the statistical analysis. B. D. wrote the first draft. All authors reviewed and approved the final version of the report and the manuscript.
