# Supplementary material for: Epidemiology, Clinical Manifestations, Treatment, and Outcome of Mucormycosis: A Review of 77 Cases From a Single Center in France
Source: Open Forum Infect Dis. 2024 Jul 23;11(8):ofae426. doi: 10.1093/ofid/ofae426 (PMC11342388; doi:10.1093/ofid/ofae426)
Supplement: ofae426_Supplementary_Data [file ofae426_supplementary_data.docx]

Supplementary appendix

[**Supplementary methods** 2](#_Toc132476123)

[*Data were collected from the HER* 2](#_Toc132476124)

[*Lab procedures* 2](#_Toc132476125)

[**EORTC/MSG diagnostic criteria** 4](#_Toc132476127)

[**Table S1. Classification of the 77 mucormycosis cases according to EORTC/MSG criteria** 6](#_Toc132476126)

[**Table S2. Laboratory methods used for the mycological diagnosis of the 77 mucormycosis cases** 7](#_Toc132476126)

# **Supplementary methods 1**

# *Data were collected from the HER*

Age, gender

Underlying condition:

- hematologic stem cell transplantation: date of transplantation, conditioning regimen, Graft Versus Host (GVH) disease, immunosuppressive regimen, haematologic status at mucormycosis diagnosis (complete remission, aplasia, number of days of aplasia, relapse, refractory disease)
- burn patients (burn type, % of burned surface)
- trauma
- solid organ transplant recipients
- diabetes mellitus
- corticosteroids exposure: presence at diagnosis and also if corticosteroids dose was above 0.3 mg/kg/day for more than 3 weeks
- prior history of other fungal infection,
- prior antifungal treatment before mucormycosis diagnosis (all antifungals prescribed within 6 weeks before mucormycosis diagnosis (prophylaxis, empiric, preemptive, curative treatments)

Patient signs and symptoms and radiological imaging at mucormycosis diagnosis

Pathology and mycologic diagnosis : mucormycosis location, type of mycological diagnosis (direct examination, culture, qPCR), signs of invasion on pathology examination, presence of a concurrent fungal or bacterial infection.

Antifungal treatment for mucormycosis episode (type, dose and treatment duration for all antifungal treatments

Surgical treatments and other measures (tapering of immunosuppressive treatment, treatment of diabetes)

# *Lab procedures*

For direct microscopic examination (DE), clinical samples were studied in chlorazol black, and calcofluor plus 10% potassium hydroxide (BD Biosciences) and considered positive for Mucormycosis when showing typical irregular, large, 90° branched, pauci septated hyphae. They were cultured on Sabouraud dextrose agar with gentamycin and chloramphenicol (Biorad) at 30°C and 37°C. All positive cultures for mucormycosis were sent to the French National Reference Center for Invasive Mycoses & Antifungals where the purity was verified and identification to the species level performed using phenotypic and molecular identification.

When undertaken, biopsies were processed in the mycology laboratory in a sterile environment and cultured on Sabouraud dextrose agar with gentamycin and chloramphenicol (Biorad) at 30°C and 37°C, using the same method for DE, and mucormycosis identification when the culture was positive. Also, when available, biopsies were studied in anatomopathology with Grocott and PAS stainings. According to the newly 2019 revised definitions of invasive fungal disease of the European Organization for Research and Treatment of Cancer/Mycosis Study Group (EORTC/MSG) (Donnelly CID 2019), histology was considered as proven mycormycosis when showing non septate, large ribbon-like hyphae, with vessel occlusion, with identification of mucormycosis by culture or PCR on tissue. Otherwise if non septate, large ribbon-like hyphae, with vessel occlusion were observed without identification of mucormycosis by culture or PCR on tissue, the case was declared as proven IFI suggesting mycormycosis.

-Detection of Mucorales DNA using qPCR in serum, on clinical samples and on fresh and frozen tissue samples was made using a previously described technique (L Millon, CID 2013).

# **Supplementary appendix 2 : 2019 EORCT/MSG diagnostic criteria**
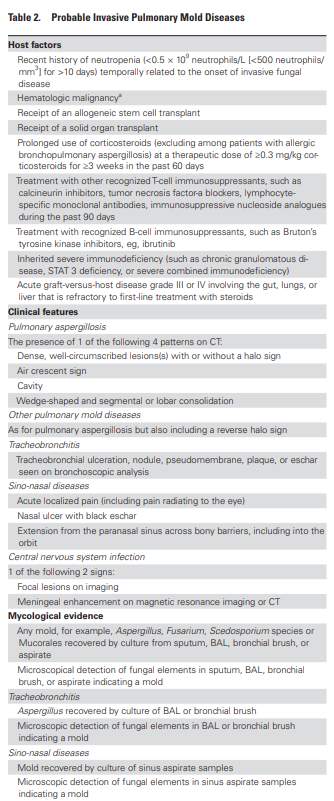

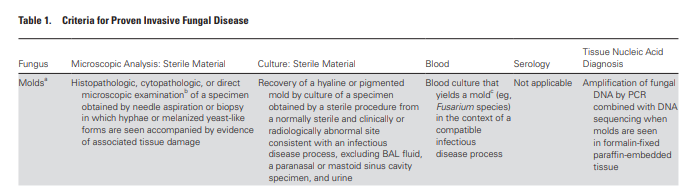


# **Table S1. Classification according to EORTC/MSG criteria of the 77 mucormycosis cases**

| **77 mucormycosis cases :** |  | **Classification according EORTC/MSG criteria** |
| --- | --- | --- |
| 45 diagnoses made by biopsies and/or puncture of sterile site : **proven IFI** | - 32 culture positive  - 8 culture negative with qPCR positive on biopsy  - 5 proven IFI suggesting mucormycosis | -**40 proven mucormycosis**  **-5 proven IFI suggesting mucormycosis** |
| 32 diagnoses without biopsies and/or puncture of sterile site | **- presence of host factor: 21 patients**  -clinical features : n: 21  -mycological evidence :  -direct exam and/or culture : 12  -only qPCR in serum or BAL or pleural liquid : 9  - **absence of host factor (ie patients with diabetes, burns, solid cancer) : 11 patients** :  11 putative mucormycosis | - **presence of host factor: 21 patients**   - **+clinical features + mycological evidence : 12/21 probable mucormycosis** - clinical features + diag by qPCR : **9/21 putative mucormycosis**   - **absence of host factor :11 putative mucormycosis** |
| **Total :** |  | **- 40 proven mucormycosis**  **- 5 proven IFI suggesting mucormycosis**  **- 12 probable mucormycosis**  **- 20 putative mucormycosis** |

# **Table S2. Laboratory methods used for the mycological diagnosis of the 77 mucormycosis cases**

| **77 mucormycosis cases :** | **Location of the samples used for mucormycosis diagnosis** | **Mucormycosis identification** |
| --- | --- | --- |
| **45 diagnoses made by biopsies and/or puncture of sterile site** | - 7 pleuropulmonary/tracheobronchial  - 17 sinus/mastoidal  - 15 cutaneous  - 4 liver  - 1 colon  - 1 kidney | - 32 culture positive  **- 8 culture negative with qPCR positive on biopsy**  - 5 proven IFI suggesting mucormycosis * |
| **32 diagnoses without biopsies** | - 22 pleuropulmonary  - 1 palatine swab  - 9 cutaneous | - 16 direct examination positive:  -12 culture +  - **4 qPCR + in serum****  - 16 direct examination negative :  -5 culture +  - **10 qPCR + in serum*****  **- 1 qPCR + in pleural liquid** |
| **Total :**  -72 mucormycosis cases identified by culture and/or qPCR  -5 cases with proven IFI suggesting mycormycosis on biopsy |  | - 49 positive cultures :  - 24 *Rhizopus*  - 13 *Mucor* (12 *M. circinelloides*, 1 *M. indicus*)  - 1 culture positive for both *Mucor* and *Rhizopus*  - 6 *Lichteimia*  - 2 *Rhizomucor*  - 2 *Cunninghamella*  - 1 *Syncephalastrum*  - Among the 28 negative or unavailable cultures, 23 diagnoses by qPCR :  - 9 qPCR *Mucor/Rhizopus,*  - 4 qPCR *Lichteimia*  - 10 qPCR *Rhizomucor* |

Direct examination positive: non septate large ribbon-like hyphae seen on sample

* Direct examination on biopsy showing non septate large ribbon-like hyphae with vessel occlusion without positive culture and/or qPCR

** and also 2 qPCR + in sputum

*** and also 4 qPCR + in sputum and Broncho Alveolar Lavage
